# A variation in *KCNQ1* gene is associated with repaglinide efficacy on insulin resistance in Chinese Type 2 Diabetes Mellitus Patients

**DOI:** 10.1038/srep37293

**Published:** 2016-11-18

**Authors:** Xueyan Zhou, Jing Zhu, Zejun Bao, Zhenhai Shang, Tao Wang, Jinfang Song, Juan Sun, Wei Li, Temitope Isaac Adelusi, Yan Wang, Dongmei Lv, Qian Lu, Xiaoxing Yin

**Affiliations:** 1 Jiangsu Key Laboratory of New Drug Research and Clinical Pharmacy, Xuzhou Medical University, Xuzhou, People’s Republic of China; 2 Department of Pharmacy, the Affiliated Hospital of Xuzhou Medical University, Xuzhou, People’s Republic of China; 3 Department of Endocrinology, the Affiliated Hospital of Xuzhou Medical University, Xuzhou, People’s Republic of China

## Abstract

Repaglinide is an insulin secretagogue that often exhibits considerable interindividual variability in therapeutic efficacy. The current study was designed to investigate the impact of *KCNQ1* genetic polymorphism on the efficacy of repaglinide and furthermore to identify the potential mechanism of action in patients with type 2 diabetes. A total of 305 patients and 200 healthy subjects were genotyped for the *KCNQ1* rs2237892 polymorphism, and 82 patients with T2DM were randomized for the oral administration of repaglinide for 8 weeks. HepG2 cells were incubated with repaglinide in the absence or presence of a KCNQ1 inhibitor or the pcDNA3.1-hKCNQ1 plasmid, after which the levels of Akt, IRS-2 and PI(3)K were determined. Our data showed that repaglinide significantly decreased HOMA-IR in patients with T2DM. Furthermore, the level of HOMA-IR was significantly reduced in those patients with CT or TT genotypes than CC homozygotes. The KCNQ1 inhibitor enhanced repaglinide efficacy on insulin resistance, with IRS-2/PI(3)K/Akt signaling being up-regulated markedly. As in our clinical experiment, these data strongly suggest that *KCNQ1* genetic polymorphism influences repaglinide response due to the pivotal role of *KCNQ1* in regulating insulin resistance through the IRS-2/PI(3)K/Akt signaling pathway. This study was registered in the Chinese Clinical Trial Register on May 14, 2013. (No. ChiCTR-CCC13003536).

Type 2 diabetes mellitus (T2DM) is a multifactorial disease resulting from complex interactions between multiple genes and environmental factors^1^. T2DM is characterized by chronic hyperglycemia, which is known to be caused by insufficient insulin secretion and insulin resistance (IR)^2^. Oral antidiabetic drugs are among the most widely prescribed medications for lowering blood glucose levels.

Repaglinide, a short-acting insulin secretagogue, mimics early phase insulin release and provides improved control of postprandial glucose levels^3^. The insulinotropic action of repaglinide is mediated by adenosine triphosphate (ATP)-dependent potassium channels (K_ATP_) in the pancreatic β-cell membrane^4^. In addition, several studies have demonstrated that repaglinide can also improve insulin sensitivity and decrease homeostasis model assessment insulin resistance (HOMA-IR) in patients with T2DM^5^,^6^. Although it has been widely used clinically and displays excellent safety and efficacy, the response to repaglinide varies among individuals, and genetic variation within KQT-like subfamily, member 1 (*KCNQ1*) has been suggested^7^.

*KCNQ1* encodes a voltage-gated K^+^ channel with six transmembrane regions and is required for the repolarization phase of the cardiac action potential^8^. A meta-analysis in East Asians confirmed that *KCNQ1* rs2237892 was the polymorphism most strongly associated with T2DM susceptibility^9^. In addition, *KCNQ1* also influences the efficacy of repaglinide; however, the underlying mechanisms remain unclear^10^,^11^,^12^. A clinical study demonstrated that diabetic patients with the *KCNQ1* rs2237892 T allele were more likely to have a positive response to repaglinide, while patients with the *KCNQ1* rs2237892 C allele exhibited larger increases in HOMA-IR^13^. Another study indicated that patients with T2DM with the *KCNQ1* rs2237892 T allele were more likely to have a positive response to repaglinide in terms of postprandial plasma glucose (PPG) levels rather than HOMA-IR levels^14^. Thus, the relationship between *KCNQ1* rs2237892 polymorphism and repaglinide efficacy on IR needs to be re-evaluated.

IR occurs extensively in hepatocytes, where insulin signal transduction and glucose metabolism also thrive^15^. The insulin signaling pathway [insulin receptor substrate protein-2 (IRS-2) - phosphatidylinositol 3-kinase [PI(3)K] - protein kinase B (AKT) axis] has been implicated in hepatic IR^16^,^17^,^18^,^19^,^20^. It is unknown whether *KCNQ1* affects the insulin signaling pathway, and whether IR is the underlying mechanism through which *KCNQ1* rs2237892 polymorphism influences repaglinide response in patients with T2DM is also unclear.

In this study, we determined the *KCNQ1* variant genotypes and the efficacy of repaglinide in newly diagnosed Chinese type 2 diabetes patients who had been treated with repaglinide for 8 weeks, investigated the association between *KCNQ1* rs2237892 polymorphism and repaglinide therapeutic efficacy, and further explored the correlation between this association and the IRS-2/PI3K/Akt signaling pathway.

## Results

### Clinical and biochemical characteristics of subjects

Samples from 305 patients with T2DM and 200 healthy controls were used to determine genotypes with regard to the *KCNQ1* rs2237892 polymorphism. The clinical characteristics of all the subjects in this study are summarized in Table 1. No significant difference was found between the two groups for sex distribution, age, or plasma concentrations of high-density lipoprotein cholesterol (HDL-c). However, there were significant differences between the patients with T2DM and healthy controls in body mass index (BMI) (*P* < 0.001), waist to hip ratio (WHR) (*P* < 0.001), fasting plasma glucose (FPG) (*P* < 0.001), triglycerides (TG) (*P* < 0.001), total cholesterol (TC) (*P* < 0.001), and low-density lipoprotein cholesterol (LDL-c) (*P* < 0.001).

### Genotyping analysis and allele frequencies

The allele frequencies of the *KCNQ1* rs2237892 polymorphism in patients with T2DM and healthy controls are given in Table 2. The genotype distribution was consistent with Hardy-Weinberg equilibrium (*P* > 0.05). The frequency of the *KCNQ1* rs2237892 C allele was higher in the patients with T2DM than in the control group (70.82% vs 61.50%, respectively, *P* < 0.01).

### Comparison of the baseline parameters of *KCNQ1* rs2237892 (C/T) genotypes among patients with T2DM

The baseline clinical characteristics of the 305 patients with T2DM were measured before repaglinide therapy. There was no significant difference in age, BMI, WHR, FPG, PPG, hemoglobin A1c (HbA1c), TG, TC and HDL-c among different genotypic groups. However, the *KCNQ1* rs2237892 polymorphism was associated with levels of fasting insulin (FINS), postprandial serum insulin (PINS) and HOMA-IR and HbA1c (%); individuals with the CC genotype had noticeably lower FINS (*P* < 0.05), PINS (*P* < 0.01), and HOMA-IR (*P* < 0.05) with higher HbA1c (%) (*P* < 0.05) levels than those individuals with other genotypes (Table 3).

### Effects of *KCNQ1* rs2237892 polymorphisms on therapeutic efficacy of repaglinide in patients with T2DM

To exclude the impact of *OATP1B* and *CYP2C8* genetic polymorphisms on the response to repaglinide, 82 patients with T2DM (48 men and 34 women) with the same *OATP1B1* 521TT and CYP2C8*3 139 Arg genotypes but different *KCNQ1* rs2237892 genotypes (44 patients with a CC genotype, 38 patients with a CT/TT genotype) were randomly selected to participate in the therapeutic efficacy study. All 82 patients with T2DM were treated with 3 mg repaglinide daily for 8 weeks. As indicated in Table 4, repaglinide treatment significantly increased FINS (*P* < 0.01) and PINS (*P* < 0.001), but remarkably decreased FPG (*P* < 0.001), PPG (*P* < 0.001), HOMA-IR (*P* < 0.01), HbA1c (%) (*P *< 0.001) and TC (*P* < 0.05). Furthermore, the levels of FPG, PPG, HOMA-IR, HbA1c (%) and LDL-c were significantly reduced in those patients with CT or TT genotypes compared with CC homozygotes, and those patients with a CT/TT genotype experienced a significant increase in PINS versus CC carriers (Table 5).

### KCNQ1 regulates insulin signal transduction in HepG2 cells

**Figure 1:**
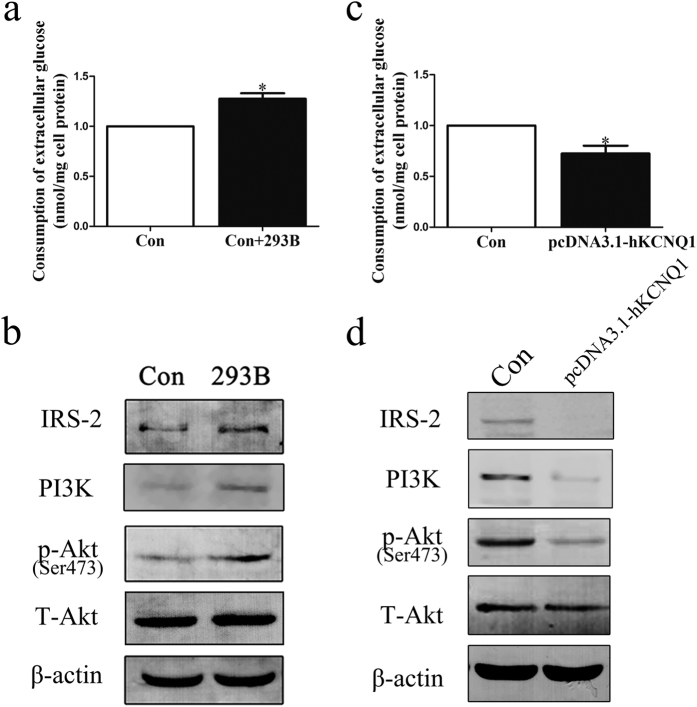
Effects of KCNQ1 on insulin signaling in HepG2 cells. (**a**,**c)** Histograms showing the consumption of extracellular glucose. (**b**) HepG2 cells were treated with chromanol 293B (10^−1^ mM) for 12 h. A representative western blot of IRS-2, PI(3)K, p-Akt (ser473), Akt, and β-actin levels is shown. (**d**) HepG2 cells were transfected for 6 h with a mixture of the plasmid and PEI. A representative western blot of IRS-2, PI(3)K, p-Akt (ser473), Akt, and β-actin levels is shown. The scanning densitometry results (n = 3) are expressed as arbitrary units. Columns and bars represent the mean ± SEM. *p < 0.05, vs Con.

To further validate the mechanism by which KCNQ1 regulates repaglinide therapeutic efficacy, relationship between KCNQ1 expression and the IRS-2/PI(3)K/Akt signaling pathway was investigated. As shown in Fig. 1a, a KCNQ1 inhibitor (chromanol 293B) significantly increased the ratio of glucose uptake in HepG2 cells. In addition, the expression of p-Akt, IRS-2 and PI(3)K were up-regulated markedly after chromanol 293B treatment (Fig. 1b). Using RT-PCR and western blotting analysis, we found that the expression of KCNQ1 mRNA and the KCNQ1 protein levels were increased significantly in pcDNA3.1-hKCNQ1 transfected cells (see Supplementary Fig. S1). As predicted, the overexpression of KCNQ1 hindered glucose metabolism and diminished IRS-2/PI(3)K/Akt signaling in HepG2 cells (Fig. 1c,d). These results indicated that KCNQ1 has a pivotal role in insulin signal transduction through the IRS-2/PI(3)K/Akt pathway.

### A KCNQ1 inhibitor activates the IRS-2/PI(3)K/Akt signaling pathway in insulin-resistant HepG2 cells

**Figure 2:**
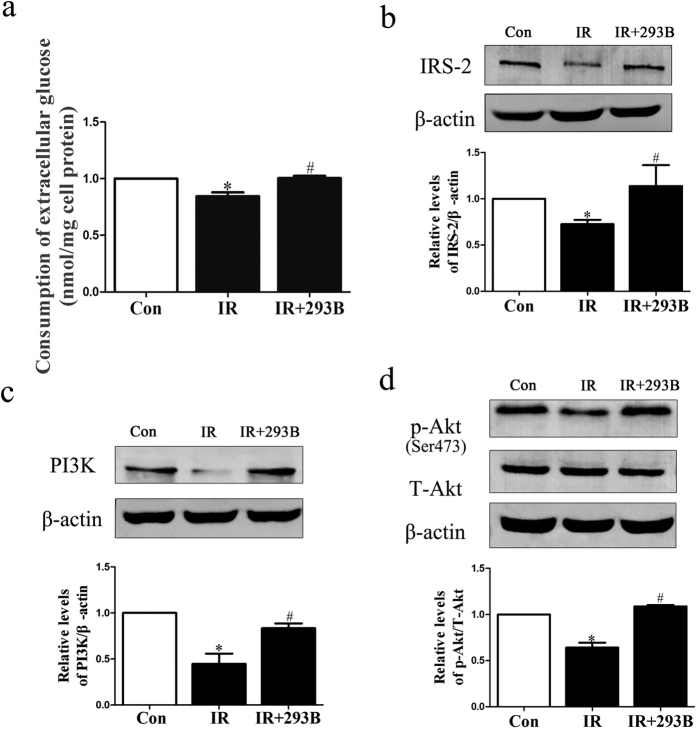
Effect of KCNQ1 inhibitor on insulin signal transduction in the IR model. (**a**) Histograms showing quantification of the consumption of extracellular glucose. (**b**–**d)** HepG2 cells were cultured in DMEM (25 mM glucose). Serum-starved cells were treated with 10^−3^ mM insulin for 6 h before exposure to chromanol 293B (293B, 10^−1^ mM) for 12 h. The extracts were immunoblotted with anti-IRS-2, anti-PI(3)K and anti-phospho-Akt. The scanning densitometry results (n = 3) are expressed as arbitrary units. Columns and bars represent the mean ± SEM. *p < 0.05, Con vs IR; ^#^*p* < 0.05, IR vs IR + 293B.

An insulin-resistant HepG2 cell model was established according to a previously described method^21^ with the slight modification of using medium containing 10^−9^ mol/L insulin. As shown in Fig. 2a, hyperinsulinemia induced a significant decrease in glucose intake in the IR group, indicating that the IR model has been successfully established. When treated with chromanol 293B, the specific inhibitor of KCNQ1, the intracellular glucose concentration returned to a normal level (Fig. 2a). Furthermore, we also observed that chromanol 293B exposure reversed the decrease in IRS-2, PI(3)K and phospho (Ser473)-Akt/total Akt protein expression following the exposure of HepG2 cells to hyperinsulinemia (Fig. 2b–d). These data suggested that KCNQ1 can regulate insulin signal transduction by activating the IRS-2/PI(3)K/Akt signaling pathway.

### KCNQ1 controls repaglinide therapeutic efficacy in insulin resistance

**Figure 3:**
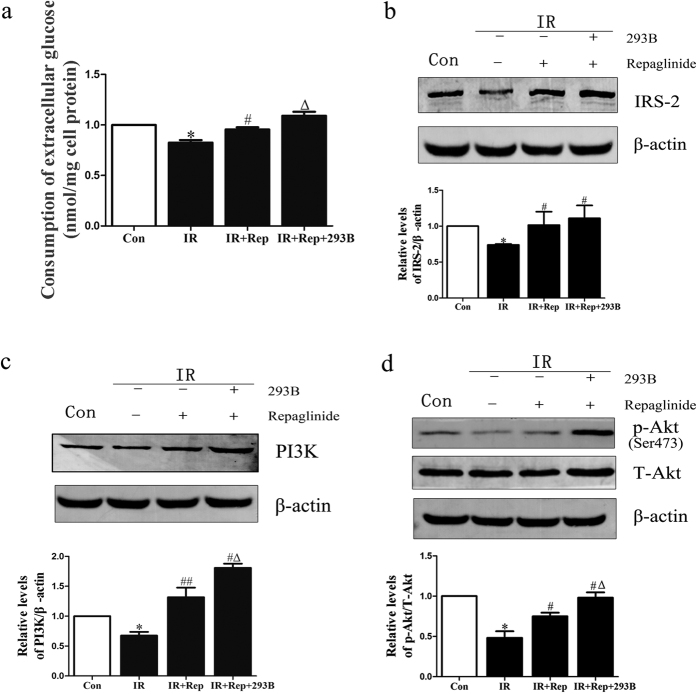
KCNQ1 controls repaglinide therapeutic efficacy through the IRS-2/PI(3)K/Akt signaling pathway. HepG2 cells were cultured in DMEM (25 mM glucose). Serum-starved cells were treated with 10^−3^ mM insulin for 6 h before exposure to repaglinide (Rep, 10^−1^ mM) with or without chromanol 293B (293B, 10^−1^ mM) for 12 h. (**a**) Enzymatic methods were used to assay for glucose. (**b**–**d)** Extracts were immunoblotted with anti-IRS-2, anti-PI(3)K and anti-phospho-Akt. The scanning densitometry results (n = 3) are expressed as arbitrary units. Columns and bars represent the mean ± SEM. *p < 0.05, Con vs IR; ^#^*p* < 0.05, IR vs IR+Rep; ^△^*p* < 0.05 IR + Rep vs IR + Rep + 293B.

To deeply evaluate the effect of KCNQ1 on repaglinide efficacy, we examined the influence of KCNQ1 expression on repaglinide therapeutic efficacy using the IRS-2/PI(3)K/Akt signaling pathway. As shown in Fig. 3a, the exposure of HepG2 cells to chronic hyperinsulinism resulted in a decrease in glucose uptake, while repaglinide exposure increased the intake of glucose in IR model cells, and the efficacy of repaglinide was significantly enhanced after treatment with chromanol 293B. Furthermore, we evaluated the insulin signal transduction pathway in HepG2 cells treated with repaglinide with or without chromanol 293B. Hyperinsulinism induced a considerable decrease in IRS-2, PI(3)K expression and the phosphorylation of Akt in insulin-resistant cells. Treatment with repaglinide prevented this effect. Moreover, the combined therapy (chromanol 293B + repaglinide) enhanced repaglinide efficacy and increased IRS-2, PI(3)K expression and phosphorylation of Akt (Fig. 3b–d). These data indicate that KCNQ1 influenced repaglinide therapeutic efficacy through the IRS-2/PI(3)K/Akt signaling pathway.

## Discussion

In the present study, an association between *KCNQ1* rs2237892 and repaglinide efficacy was observed with respect to HOMA-IR in newly identified Chinese type 2 diabetes patients. Most importantly, we also observed that *KCNQ1* plays a pivotal role in determining insulin signal transduction through the IRS-2/PI(3)K/Akt signaling pathway.

Several pharmacogenomic studies have demonstrated that variations in the genes involved in the pharmacokinetics or pharmacodynamics of repaglinide are associated with the drug response^4^. Polymorphisms in the genes involved in drug metabolism, such as cytochrome P450 (CYP) 2C8^22^ and organic anion-transporting polypeptide 1B1 (OATP1B1) may influence the efficacy of repaglinide and the incidence of adverse effects^23^. To exclude the impact of *OATP1B* and *CYP2C8* genetic polymorphisms on the response to repaglinide, participants with the same *OATP1B1* 521TT and CYP2C8*3 139 Arg genotypes but different *KCNQ1* rs2237892 genotypes were randomly selected. Consistent with the results in Korean and Malaysian subgroups, we determined in our study that the frequency of the C allele in *KCNQ1* rs2237892 was significantly higher in patients with T2DM than in healthy controls^24^ (Table 2). With respect to repaglinide efficacy, patients with the CC genotype exhibited lower IR at baseline (HOMA-IR = 4.24) than individuals with the CT/TT (HOMA-IR = 5.67) genotypes (P = 0.046) (Table 3). This is probably due to the lower FINS and more serious deficiency in pancreatic β-cell function in CC individuals (P = 0.014) (Table 3). Despite a more serious IR at baseline, CT/TT individuals responded better to repaglinide treatment in terms of decreasing HOMA-IR than CC individuals. The ∆ values for HOMA-IR (P = 0.041) at pre and post treatment were significantly different between different genotypes (Table 5). The underlying mechanism responsible for these findings is not known, but the poor repaglinide response in patients with the risk-associated C allele of *KCNQ1* rs2237892 might possibly result from the specific effects of the C allele on IR.

Repaglinide is a fast-acting insulin secretagogue that initiates insulin secretion by closing ATP-dependent potassium channels. It helps mimic the early rise in insulin concentration, which ensures its primary efficacy in normalizing postprandial glycemic elevation^25^. In addition, reductions in FPG and PPG levels were also observed after repaglinide treatment^26^. However, it has been reported that neither patients with cardiac arrhythmia caused by *KCNQ1* mutations nor *Kcnq1*-null mice demonstrate hyperglycemia or glucose intolerance^27^,^28^, whereas *KCNQ1* knockdown in human islets does not alter insulin secretion^29^. Thus, variation in the *KCNQ1* gene may not be associated with repaglinide effects on insulin secretion.

*KCNQ1* encodes a voltage-gated K^+^ channel with six transmembrane regions that is involved in the repolarization of the action potential in cardiac muscle^30^,^31^,^32^. A GWAS in the East Asian population identified *KCNQ1* as a susceptibility gene for T2DM^33^. This association between *KCNQ1* and T2DM has been replicated in Caucasian^34^ and Chinese populations^35^. Mutations in *KCNQ1* have been associated with cardiac diseases such as long-QT syndrome and familial atrial fibrillation^36^,^37^. However, *KCNQ1* is also expressed in other tissues, including islet cells, liver, and skeletal muscle^38^. A recent study observed a striking increase in insulin sensitivity in the *Kcnq1* knockout mouse model, thereby raising the possibility that *Kcnq1* may be a novel element that affects insulin sensitivity^31^. Yu *et al*.^13^ performed a randomized pharmacogenetics study to evaluate the possible impact of *KCNQ1* polymorphism on the efficacy of repaglinide in 2011. They found that rs2237892 TT homozygous individuals exhibited lower PPG levels and significantly higher cumulative attainment rates of the target PPG levels than the C allele carriers. Moreover, the patients carrying more C alleles at rs2237892 or rs2237895 displayed larger increases in both fasting insulin levels and HOMA-IR. Here, larger samples were investigated to replicate previous findings. In this study, 82 patients with T2DM were randomized to receive orally administered repaglinide. After 8 consecutive weeks of therapy, repaglinide significantly decreased HOMA-IR in patients with T2DM, with CC homozygotes exhibiting a higher increase in HOMA-IR (P < 0.05) than in *KCNQ1* CT/TT carriers. These results verified that *KCNQ1* single nucleotide polymorphisms (SNPs) have an impact on the efficacy of repaglinide in improving insulin sensitivity. Nevertheless, the underlying mechanism remains unknown.

To fully delineate the role of *KCNQ1* on repaglinide efficacy and its related pathways in IR, we established an insulin-resistant model in HepG2 cells. The *KCNQ1* locus harbors at least two independent regions of association with type 2 diabetes risk (intron 10 and intron 15), both acting through impaired islet function^39^. Recently, evidences showed that *KCNQ1* SNPs influenced gene expression or function through the introduction or removal of a CpG site via differential DNA methylation and subsequently had an impact on the pathogenesis of type 2 diabetes^40^. Hence, we assumed that the regulation of *KCNQ1* gene expression in cells could mimic the polymorphism and the functional variations of *KCNQ1* in the clinic. Insulin signaling is initiated through its receptor tyrosine kinase, IRS-2, leading to phosphatidylinositol 3-kinase activation (PI3K), phosphatidylinositol 3,4,5-triphosphate generation and Akt activation^41^. In hepatocytes, chronic insulin infusion induces IR by impairing glucose uptake and the IRS-2/PI(3)K/Akt signaling pathway, which were also identified as critical nodes in the regulation of hepatocyte insulin sensitivity^42^. In this study, we found that a KCNQ1 selective inhibitor (chromanol 293B) increased the uptake of extracellular glucose and activated the IRS-2/PI3K/Akt pathway in normal and IR HepG2 cells, indicating that KCNQ1 has a pivotal role in regulating insulin signal transduction pathways under both physiological and pathological conditions. To further evaluate whether KCNQ1 is involved in repaglinide efficacy, a cellular study of repaglinide efficacy on glucose uptake was carried out. In the IR cell model, we observed that the KCNQ1 inhibitor enhanced repaglinide efficacy in IR, with IRS-2/PI(3)K/Akt signaling being up-regulated markedly. Similar to the results of the clinical experiment, these data strongly suggested that repaglinide can increase hepatic insulin sensitivity, which can be further enhanced by the inhibition of KCNQ1. To the best of our knowledge, this is the first study to elucidate the role of IRS-2/PI(3)K/Akt in the influence of *KCNQ1* polymorphisms on repaglinide therapeutic efficacy in T2DM.

There are several limitations of this study that should be noted. First, the sample size was relatively small. In this study, only 82 patients were recruited based on our criteria for newly diagnosed and untreated type 2 diabetic patients; thus, we did not have enough statistical power to detect the effect of the genetic variants on some of the parameters. Second, individual differences are the product of interactions between multiple genetic and environmental factors, which hinders determination of the most efficacious therapy or risk of adverse events for individual patients. Distinct repaglinide responses were observed and may result from various genetic factors; perhaps other susceptible genetic variations are also involved in repaglinide therapeutic efficacy. Next, genetic variation has multiple effects on the proteome. It may influence the expression level of protein, modify amino acid sequences through SNPs, transform the occurrence of allelic variants, or change the alternative splicing (ASP) events^43^. More detailed research is required to elucidate the mechanism by which *KCNQ1* rs2237892 polymorphism influences the IRS-2/PI(3)K/Akt signaling pathway and repaglinide response in patients with T2DM.

In summary, we herein present novel data proving that *KCNQ1* polymorphisms influence T2DM risk and clinical response to repaglinide by regulating the IRS-2/PI(3)K/Akt signaling pathway. Our findings provide new insight into better strategies to predict therapeutic efficacy and control blood glucose in patients with T2DM with different *KCNQ1* genotypes. Furthermore, our data also suggest that *KCNQ1* may be a valuable target for anti-diabetic therapy and useful for medical optimization in clinical settings. However, more detailed genetic and functional investigations are needed to examine the effects of *KCNQ1* variants on repaglinide therapy to provide more experimental evidence for patient-tailored therapy.

## Methods

### Cohort

The study of the *KCNQ1* rs2237892 polymorphism was conducted on 305 patients with T2DM (165 men and 140 women; age 48.93 ± 11.89 years) with no history of prior antidiabetic medications and 200 healthy controls (99 men and 101 women; age 50.07 ± 6.42 years) from the Affiliated Hospital of Xuzhou Medical University, Xuzhou, China. The patients with T2DM were enrolled from the Department of Endocrinology, whereas the healthy subjects were enrolled from the Health Screening Center of the Affiliated Hospital of Xuzhou Medical University. All subjects were evaluated through medical history, physical examination and routine clinical laboratory tests. Detailed information, inclusion criteria and exclusion criteria for this study population have been described previously^44^,^45^. In brief, T2DM was diagnosed according to the 1999 World Health Organization criteria on the basis of fasting plasma glucose (FPG) > 7.0 mmol/L or postprandial plasma glucose (PPG) levels > 11.1 mmol/L. The healthy subjects should have no history of T2DM, no symptoms of T2DM include increased thirst, increased hunger, frequent urination and unexplained weight loss. In addition, the fasting plasma glucose (FPG) of healthy subjects should be between 3.9 and 6.1 mmol/L. The inclusion criteria for all subjects were a body mass index (BMI) of 18.5–30 kg/m^2^, 30–70 years of age and no treatment in the previous 3 months with any insulin secretagogue and/or agonists or inhibitors of CYP2C8, CYP3A4 and OATP1B1. Patients with type 1 diabetes mellitus, a history of ketoacidosis, ischemic heart disease, congestive heart failure or trauma, or kidney or liver diseases were excluded from the study, as were those who were receiving insulin treatment and pregnant or lactating women. All patients received a standard diabetes curriculum with a specific focus on diet and exercise. For diet, dietitians established different nutritional plans for patients according to their age, height, weight, eating habits, and patient condition. For exercise, patients were typically advised to achieve more than 30 minutes of brisk walking after meals at least 5 days/week.

Among the 305 patients with T2DM in the study, 82 patients with T2DM (48 men and 34 women) carrying different *KCNQ1* rs2237892 genotypes and the same CYP2C8*3 139Arg and *OATP1B1* 521TT genotypes (data not shown) were randomized to receive repaglinide monotherapy (1 mg 3 times/day) for 8 consecutive weeks. To randomize the participants, a computer-generated list of random numbers was prepared by an investigator with no clinical involvement in the trial. The study was registered in the Chinese Clinical Trial Register on May 14, 2013 (No. ChiCTR-CCC13003536) and was performed in accordance with the Helsinki Declaration; the protocol was approved by the Ethics Committee of the Affiliated Hospital of Xuzhou Medical University. Written informed consent was obtained from each subject before the study.

### Clinical assessment

BP was measured twice in a supine position using a mercury sphygmomanometer after 5 min of rest; mean values were used. Weight and height were measured in lightly clad participants, and body mass index (BMI) was calculated. BMI = weight (kg)/height^2^ (m).

### Clinical laboratory tests

At the end of weeks 0 and 8 after administration of repaglinide, venous blood samples were collected after an overnight fast and 2 hours after a standard breakfast (a 100-g steamed bread meal). Estimation of glucose level was carried out using the glucose oxidase-peroxidase method and serum lipids were assessed using a Roche Cobas 8000 analyzer (Roche, Basel, Switzerland), while plasma insulin and glycated hemoglobin (HbA1c) levels were measured with electro-chemiluminescence (Roche, Shanghai, China) and high-performance liquid chromatography (HPLC) assays, respectively. The homeostasis model assessment (HOMA-IR) was calculated as follows: HOMA-IR = fasting insulin (μU/ml) × fasting glucose (mmol/l)/22.5.

### Genotyping

Genomic DNA was isolated from peripheral blood leucocytes using a SiMax Genome DNA Kit (Sbsbio, Shanghai, China) and then stored at 4 °C until use. *KCNQ1* rs2237892 polymorphisms were genotyped using polymerase chain reaction-restriction fragment length polymorphisms (PCR-RFLP) with the following primers: 5′-CTTGTGCCCTTGTCACCCAC-3′ (forward) and 5′-GGCTTCCAGCCT CCAAGCTG-3′ (reverse). The polymerase chain reaction products were digested with MspI (NEB, Beijing, China). The CYP2C8*3 Arg139Lys genotype was also detected with the PCR-RFLP assay, and the primers used were 5′-AGGCAATTCCCCAAT ATCTC-3′ (sense) and 5′-ACTCCTCCACAAGGCAGTGA-3′ (antisense). Amplified DNA was digested with BseRI (NEB, Beijing, China). *OATP1B1* T521C genotypes were detected using the amplification refractory mutation system with four primers: forward primer: 5′-AAGTAGTTAAATTTGTAATAGAAATGC-3′, reverse primer: 5′-GTAGACAAAGGGAAAGTGATCATA-3′; forward primer for wild-type genotype: 5′-GGGTCATACATGTGGATATAAGT-3′, reverse primer for mutant variants: 5′-AA GCATATTACCCATGAACG-3′.

### Cell culture

HepG2 cells were obtained from the Type Culture Collection of the Chinese Academy of Sciences (Shanghai, China) and seeded into tissue culture dishes in Dulbecco’s modified Eagle’s medium (DMEM) with high glucose plus 15% heat-inactivated fetal bovine serum and 1% penicillin/streptomycin and were placed in a humidified incubator at 37 °C and 5% CO_2_. The insulin-resistant cell model was induced using the previous method with minor modifications. HepG2 cells were allowed to attach for 12 h and were then serum-starved for 8 h. HepG2 cells were incubated with fresh medium containing 1% FBS, 10^−3^ mM insulin (Wanbang Biologic & Medicinal Co., Ltd., Jiangsu, China) for 6 h. Subsequently, the medium was exchanged with medium containing 1% FBS, 10^−6^ mM insulin and repaglinide (10^−1^ mM, Sigma) or chromanol 293B (10^−1^ mM, Sigma). Cells were incubated in this medium for 12 h.

The human pcDNA3.1-hKCNQ1 vector was constructed by Biogot (Nanjing, China) using pSP64-hKCNQ1 (Addgene, Cambridge, MA). HepG2 cells were grown in tissue culture dishes and transfected for 6 h with a mixture of the plasmid and PEI. Then, the mixture was replaced with full medium and incubated for 48 h.

### Extracellular glucose detection in HepG2 cells

A glucose uptake assay was conducted as described previously. After treatment, 10 μl of medium was used to assay for glucose using enzymatic methods with diagnostic kits (Nanjing Jiancheng Bioengineering Inst.). Data were expressed as the consumption of extracellular glucose (nmol/mg protein), which was calculated as follows: [extracellular glucose content (nmol) before – extracellular glucose content (nmol) after]/mg cell protein, which was measured using a BCA protein assay (Pierce, Rockford, Ill., USA.).

### Western blotting analysis

HepG2 cells were washed twice with ice-cold phosphate-buffered saline (PBS) and lysed in buffer containing 50 mM Tris-HCl (pH 7.6), 150 mM NaCl, 1 mM EDTA, 1% NP-40, 1 mM PMSF, 1 mM Na_3_VO_4_ and 20 mM NaF, and then sonicated. Each protein sample (60 μg) was separated by 8% sodium dodecyl sulfate polyacrylamide gel electrophoresis (SDS-PAGE) and transferred to nitrocellulose membranes. The membranes were blocked with 3% BSA for 1 h at room temperature and then incubated with different primary antibodies, including rabbit polyclonal antibodies against KCNQ1, IRS-2 (Abcam, Cambridge, USA), Akt, phospho-Akt, β-actin (Bioworld Technology, St. Louis, USA) and a mouse monoclonal antibody against PI(3)K (Abcam, Cambridge, USA), for 12 h at 4 °C, followed by incubation with secondary antibody for 2 h. The membranes were exposed to the BCIP/NBT alkaline phosphatase color developing kit (Beyotime Institute of Biotechnology, Nantong, China). The protein density was quantified using Image J software. The density of the protein of interest was compared with either β-actin or total Akt from the same specimen, and the density of treated and control samples were compared.

### Reverse transcription-polymerase chain reaction (RT-PCR) analysis for *KCNQ1*

Total RNA was extracted from HepG2 cells using Trizol (Invitrogen, Carlsbad, CA, USA) according to manufacturer’s instructions. Reverse transcription and PCR were performed as described previously with minor modifications. The RT and PCR steps were performed using a ReverTra Ace qPCR RT kit (Toyobo Co, Osaka, Japan). For cDNA amplification, 1 μl cDNA was amplified in the presence of 1 μl each of “downstream” and “upstream” primers and 12.5 μl 2 × master mix in a total volume of 25 μl. The number of PCR cycles yielding an optimal signal was established experimentally as 30 cycles for the *KCNQ1* and GAPDH transcripts. Each cycle consisted of 30 s at 94 °C for DNA denaturation, 30 s at 60 °C for primer annealing, and 1 min at 72 °C for primer extension.

Primers for PCR were synthesized by Sangon Biotech (Shanghai, China): *KCNQ1*, 5′-GCAGAGAAGTGACGGTTCCTA-3′ (forward) and 5′-TTAGCGTTCCTATTCCT ATTCCACAGC-3′ (reverse); GAPDH, 5′-GAAGGTGAAGGTCGGAGTC-3′ (forward) and 5′-GAAGATGGTGATGGGATTTTC-3′ (reverse). After the cycles, aliquots of the PCR products were resolved by electrophoresis in a 2% agarose gel, followed by Gold view^TM^ staining and visualization with UV transillumination.

### Statistical analysis

In the clinical study, data were expressed as the mean ± SD for variables with a skewed distribution, while categorical data were presented as percentages. All statistical analyses were carried out using SPSS software (version 13.0 for Windows; SPSS, Chicago, IL). Hardy-Weinberg equilibrium was assessed using the χ2 test in the study sample. A comparison of baseline characteristics in patients with T2DM and healthy controls was carried out using the independent-samples t-test. Genotype and allele frequencies were analyzed using Pearson’s χ2 test. Baseline characteristics were compared among genotypes using independent-samples t-tests. To compare the effects of repaglinide on the metabolic parameters of subjects with the various genotypes, paired-samples t-tests and independent-samples t-tests were used. Parameters with normal distributions were analyzed with the Mann-Whitney test (*P* < 0.05 was considered statistically significant).

In the experimental study, mean values ± SEM were calculated, and differences between treated and control results were compared using one-way ANOVA with a Tukey-Kramer post-test for multiple comparisons or two-tailed unpaired t tests. At least three independent samples with technical replicates were analyzed for each experiment.

## Additional Information

**How to cite this article**: Zhou, X. *et al*. A variation in *KCNQ1* gene is associated with repaglinide efficacy on insulin resistance in Chinese Type 2 Diabetes Mellitus Patients. *Sci. Rep*. **6**, 37293; doi: 10.1038/srep37293 (2016).

**Publisher’s note**: Springer Nature remains neutral with regard to jurisdictional claims in published maps and institutional affiliations.

## Supplementary Material

Supplementary Information

## Figures and Tables

**Table 1:** Clinical characteristics of patients with T2DM and healthy controls.

Parameter	Healthy controls (n = 200)	Patients with T2DM (n = 305)	*P* value
Sex			
Male	99	165	
Female	101	140	0.179^b^
Age (years)	50.07 ± 6.42	48.93 ± 11.89	0.250^a^
BMI (kg/m^2^)	23.97 ± 3.31	26.10 ± 3.54	0.000^***a^
WHR	0.81 ± 0.06	0.91 ± 0.06	0.000^***a^
FPG (mmol/l)	5.10 ± 0.43	9.91 ± 2.70	0.000^***a^
TG (mmol/l)	1.25 ± 0.42	2.57 ± 2.92	0.000^***a^
TC (mmol/l)	4.61 ± 0.74	5.27 ± 1.30	0.000^***a^
HDL-c (mmol/l)	1.41 ± 0.16	1.43 ± 0.66	0.183^a^
LDL-c (mmol/l)	2.87 ± 0.49	3.20 ± 1.07	0.000^***a^

Data are given as the mean ± SD.

^a^*P* values were determined using the Mann-Whitney test.

^b^*P* values were determined using Pearson’s χ2 test; all other *P* values were assessed using independent-samples t-tests. ^***^P < 0.001.

**Table 2:** Comparison of the distribution of *KCNQ1* rs2237892 (C/T) polymorphism frequencies between patients with T2DM and healthy controls.

Genotype	Healthy controls (n = 200) (frequency)	Patients with T2DM (N = 305) (frequency)	*P* value
*KCNQ1* rs2237892			
CC	72 (36.00%)	148 (48.52%)	
CT	102 (51.00%)	136 (44.59%)	
TT	26 (13.00%)	21 (6.89%)	0.006^**^
Alleles			
C	246 (61.50%)	432 (70.82%)	
T	154 (38.50%)	178 (29.18%)	0.002^**^

The allelic frequencies are indicated in absolute values (percentage). *P* values were determined using Pearson’s χ2 test. ^**^P < 0.01.

**Table 3:** Baseline characteristics of patients with T2DM with various *KCNQ1* rs2237892 (C/T) genotypes before the administration of repaglinide (n = 305).

Parameter	CC (n = 148)	CT/TT (n = 157)	*P* value
Sex			
Male	90	95	
Female	58	62	0.957^b^
Age (years)	49.10 ± 10.68	48.78 ± 12.93	0.812
BMI (kg/m^2^)	25.71 ± 3.22	26.47 ± 3.79	0.109^c^
WHR	0.91 ± 0.07	0.91 ± 0.06	0.901^c^
FPG (mM)	9.95 ± 2.62	9.88 ± 2.78	0.614^c^
PPG (mM)	16.52 ± 4.47	16.20 ± 4.81	0.162^c^
FINS (mU/l)	9.99 ± 6.90	13.93 ± 13.82	0.014^*^
PINS (mU/l)	37.81 ± 46.19	57.37 ± 66.45	0.002^**c^
HOMA-IR	4.24 ± 2.71	5.67 ± 4.92	0.046^*c^
HbA1c (%)	9.42 ± 1.85	9.16 ± 2.28	0.02^*c^
TG (mM)	2.49 ± 3.19	2.64 ± 2.66	0.182^c^
TC (mmol/l)	5.25 ± 1.33	5.29 ± 1.27	0.629^c^
HDL-c (mM)	1.43 ± 0.52	1.36 ± 0.45	0.128^c^
LDL-c (mM)	3.19 ± 1.12	3.21 ± 1.04	0.971^c^

Data are given as the mean ± SD.

^b^*P* values were determined using Pearson’s χ2 test.

^c^*P* values were determined using the Mann-Whitney test; all other *P* values were assessed using independent-samples t-tests. ^*^P < 0.05,^**^P < 0.01.

**Table 4:** Clinical characteristics of all patients with T2DM before and after repaglinide treatment (n = 82).

Parameter	Before	After	*P* value
FPG (mM)	10.32 ± 2.53	7.19 ± 1.56	0.000^***b^
PPG (mM)	17.38 ± 4.61	11.11 ± 3.22	0.000^***b^
FINS (mU/l)	9.10 ± 7.90	10.47 ± 8.93	0.005^**b^
PINS (mU/l)	38.99 ± 50.63	52.50 ± 54.60	0.000^***b^
HOMA-IR	3.90 ± 2.89	3.30 ± 2.87	0.002^***b^
HbA1c (%)	9. 80 ± 2.06	7.12 ± 0.99	0.000^***b^
TG (mM)	2.22 ± 1.97	1.89 ± 1.74	0.077^b^
TC (mM)	5.19 ± 1.47	4.83 ± 1.07	0.026^*b^
HDL-c (mM)	1.45 ± 0.65	1.37 ± 0.53	0.382^b^
LDL-c (mM)	3.10 ± 1.20	3.03 ± 1.02	0.653

Data are expressed as the mean ± SD. *P* values were determined using paired-samples t-tests.

^b^*P* values were determined using the Wilcoxon test. ^*^P < 0.05, ^**^P < 0.01, ^***^P < 0.001.

**Table 5:** Comparisons of differential values (post administration minus pre administration) in patients with T2DM with different *KCNQ1* rs2237892 (C > T) genotypes before and after repaglinide treatment.

Parameter	CC (n = 44)	CT/TT (n = 38)	*P* value
Sex			
Male	26	22	
Female	18	16	0.913^b^
FPG (mM)	−2.38 ± 1.90	−4.04 ± 2.85	0.003^**^
PPG (mM)	−5.79 ± 4.12	−6.83 ± 5.00	0.003^**^
FINS (mU/l)	1.83 ± 6.57	0.86 ± 4.32	0.731^c^
PINS (mU/l)	4.19 ± 50.36	24.30 ± 35.85	0.026^*^
HOMA-IR	−0.10 ± 2.72	−1.18 ± 2.05	0.041^*c^
HbA1c (%)	−2.29 ± 1.66	−3.14 ± 1.77	0.014^*c^
TG (mM)	−0.30 ± 2.20	−0.36 ± 1.55	0.622^c^
TC (mM)	−0.21 ± 1.15	−0.54 ± 1.68	0.440^c^
HDL-c (mM)	−0.12 ± 0.82	−0.03 ± 0.71	0.672^c^
LDL-c (mM)	0.14 ± 1.05	−0.30 ± 1.44	0.000^***^

Data are given as the mean ± SD.

^b^P values were determined using Pearson’s χ2 test.

^c^P values were determined using the Mann-Whitney test; all other P values were assessed using independent-samples t-tests. *P < 0.05, **P < 0.01, ***P < 0.001.
