# Supplementary material for: A variation in KCNQ1 gene is associated with repaglinide efficacy on insulin resistance in Chinese Type 2 Diabetes Mellitus Patients
Source: Sci Rep. 2016 Nov 18;6:37293. doi: 10.1038/srep37293 (PMC5114551; doi:10.1038/srep37293)
Supplement: Supplementary Information [file srep37293-s1.pdf]

Supplementary Information

A variation in *KCNQ1* gene is associated with repaglinide efficacy on insulin resistance in Chinese Type 2 Diabetes Mellitus Patients

Xueyan Zhou[a#], Jing Zhu[a#], Zejun Bao[a], Zhenhai Shang[a,c], Tao Wang[a,c], Jinfang Song[a], Juan Sun[b], Wei Li[b], Temitope Isaac Adelusi[a], Yan Wang[c], Dongmei Lv[c], Qian Lu[a*], Xiaoxing Yin[a*]

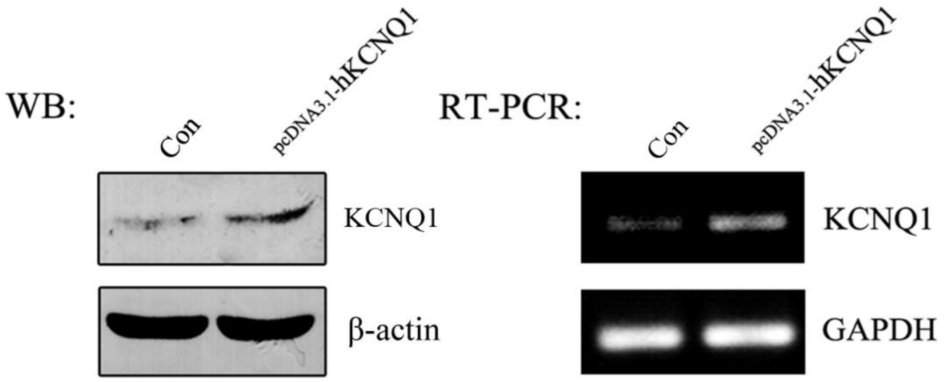

Supplementary Fig. S1 The level of KCNQ1 in HepG2 cells of controls (Con) and KCNQ1 overexpression (pcDNA3.1-hKCNQ1). HepG2 Cell line was cultured in DMEM (25 mmol/l glucose) and cells were transfected for 6 h with a mixture of the plasmid and PEI. Then the mixture was replaced by a full medium and incubated for 48 h. The lever of KCNQ1 in HepG2 cells by western blot and RT-PCR.
